# Online Survey on Twitter: A Urological Experience

**DOI:** 10.2196/jmir.2719

**Published:** 2013-10-25

**Authors:** Fabrizio Dal Moro

**Affiliations:** ^1^UrologyDepartment of Surgical, Oncological and Gastroenterological SciencesUniversity of PadovaPadovaItaly

**Keywords:** Twitter, social media, survey

After my return from the Annual Congress of the American Urological Association in San Diego (May 3-8, 2013), I would like to congratulate McKendrick et al [1] and Hanson et al [2] on their recent papers on the impact of Twitter, one of the most commonly used social media, in the medical and non-medical fields. In their elegant communications, both groups of authors stressed the role of the Internet and the Web as new methods of publicizing scientific data. Starting from the use of Twitter during a medical conference, McKendrick et al reported their first experience of using social media as support for conference organizers, highlighting the use of a Twitter stream as an integral part of the communication structure of a conference on anesthetics. Hanson et al reported a Twitter-based surveillance method for monitoring public health regarding the use and abuse of a psychostimulant drug, emphasizing the potential role of this social media in collecting data for a survey.

**Figure 1:**
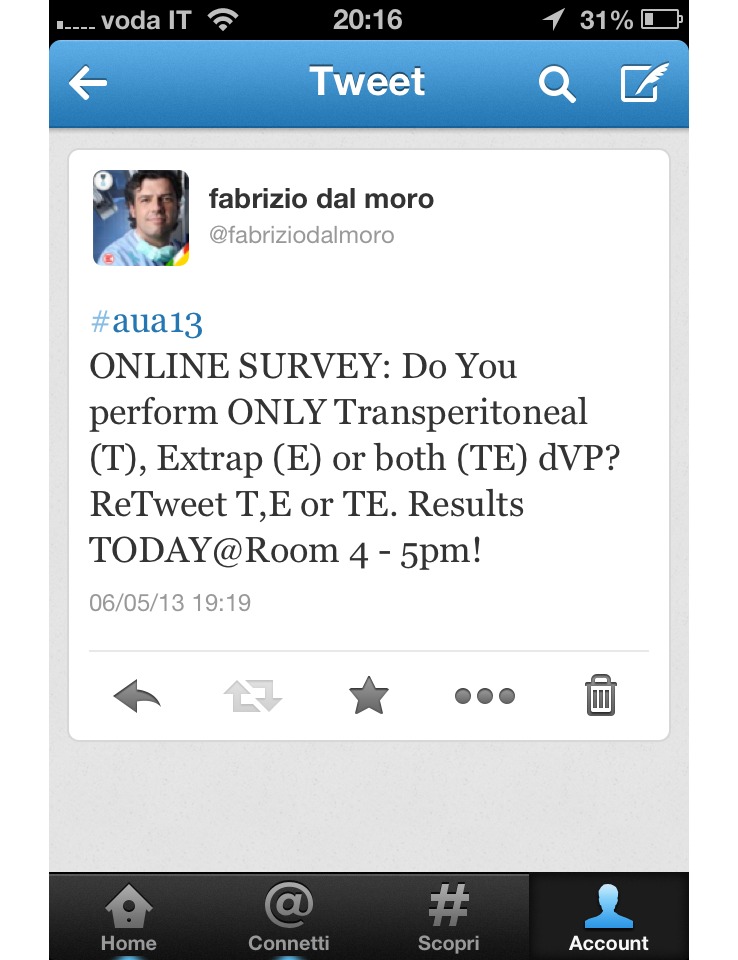
Screenshot of the Tweet.

To confirm these findings and encourage the use of these novel tools “to do science”, I would like to share a personal pioneering experiment I carried out during the Annual Congress of the European Association of Urology (March 2013, in Milan) and the recent American Congress in San Diego. During the meetings, I launched an online survey using Twitter, and posted an interactive specific urological question concerning the choice of preferred approach to robotic radical prostatectomy, the most recent and widespread surgical intervention for prostate cancer. The tweet was: “ONLINE-SURVEY: Do you perform ONLY Transperitoneal (T), ONLY Extraperitoneal (E) or BOTH approaches to dVP? (dVP= Da Vinci robotic prostatectomy) ReTweet T, E or TE” (Figure 1).

I used two specific hashtags (#EAU2013 for Milan, and #AUA2013 for San Diego).

In the course of both congresses, I received no fewer than 326 answers. Considering the absolute number of participants (approximately 18,000 in both congresses) the percentage of Tweets received (about 2%) may seem very low, but considering that it was a first "urological" experience, I think it may be significant.

Evaluating the survey results, 81 out of the 326 urologists (24.9% of those who used Twitter and retweeted me their answers!) perform both procedures. In 165 cases (50.6%), the preferred approach to robotic prostatectomy is transperitoneal.

I presented the results of this survey during my podium lecture in San Diego.

Obviously, my study is not a scientific survey without biases, but it did reveal the spread of social media, to the scientific community too.

In my opinion, this simple experiment not only confirms considerations about the use of social media by these authors, but also helps all of us to appreciate the fact that Twitter, like Facebook, Google+, Linkedin and all the other weblinked social tools, is not limited to young people “chatting about love or friends”, but are novel instruments which can be used to even greater scientific advantage.
